# Unveiling the ``vessel-inside-vessel'' phenomenon: A rare case of extensive portal vein tumor thrombosis in hepatocellular carcinoma

**DOI:** 10.1016/j.radcr.2025.02.068

**Published:** 2025-03-15

**Authors:** Mohamed Fadil, Hatim Essaber, Asaad El Bakkari, Hounayda Jerguigue, Sanae Amalik, Youssef Omor, Rachida Latib

**Affiliations:** Radiology department, National Oncology Institute, University Hospital Center Ibn Sina, Rabat, Morocco

**Keywords:** HCC, Portal vein, Metastasis, CT, US

## Abstract

Carcinomas often metastasize to distant organs by detaching from the primary tumor and entering the bloodstream. These circulating tumor cells are transported to sites such as lung capillaries and subsequently disseminated through systemic circulation. However, hepatocellular carcinoma (HCC) rarely metastasizes to distant organs, even in advanced stages. Instead, HCC frequently leads to intravascular and intrahepatic parenchymal metastases. Portal vein thrombosis (PVT), present in 10%-40% of HCC patients at diagnosis and 35%-44% at death or liver transplant, is a significant prognostic marker, associated with worse outcomes and reduced survival. Patients with PVT demonstrate markedly shorter overall survival compared to those without PVT, with main portal vein thrombosis indicating the poorest prognosis. Here, we report an unusual case of an enormous portal metastasis from liver HCC, characterized by a ``vessel-inside-vessel'' appearance.

## Case report

A 63-year-old woman with a medical history of familial hyperlipidemia underwent a routine health examination, including liver ultrasonography (US), for hyperlipidemia monitoring. This revealed multiple hypodense hepatic lesions. Physical examination and laboratory investigations were unremarkable, prompting further imaging with an abdominal computed tomography (CT) scan.

Contrast-enhanced abdominal CT was performed during the arterial, portal, and delayed phases. Multiple hypodense hepatic lesions with vivid enhancement in the arterial phase and washout in the delayed phase were observed, alongside areas of central necrosis. The enhancement pattern was consistent with hepatocellular carcinoma.

**Fig. 1:**
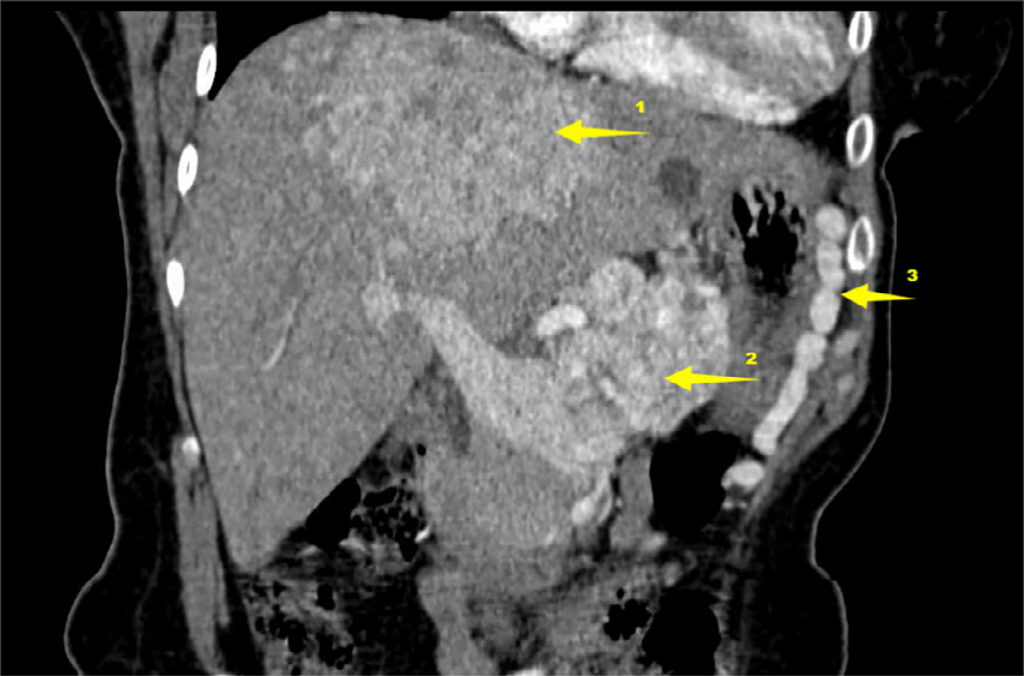
Abdominal injected CT on coronal plane. 1 liver tumor. 2 tumoral thrombosis inside portal vein containing multiple small hyperdense vessel, giving the vessel in vessel appearance. 3 collateral circulation.

**Fig. 2:**
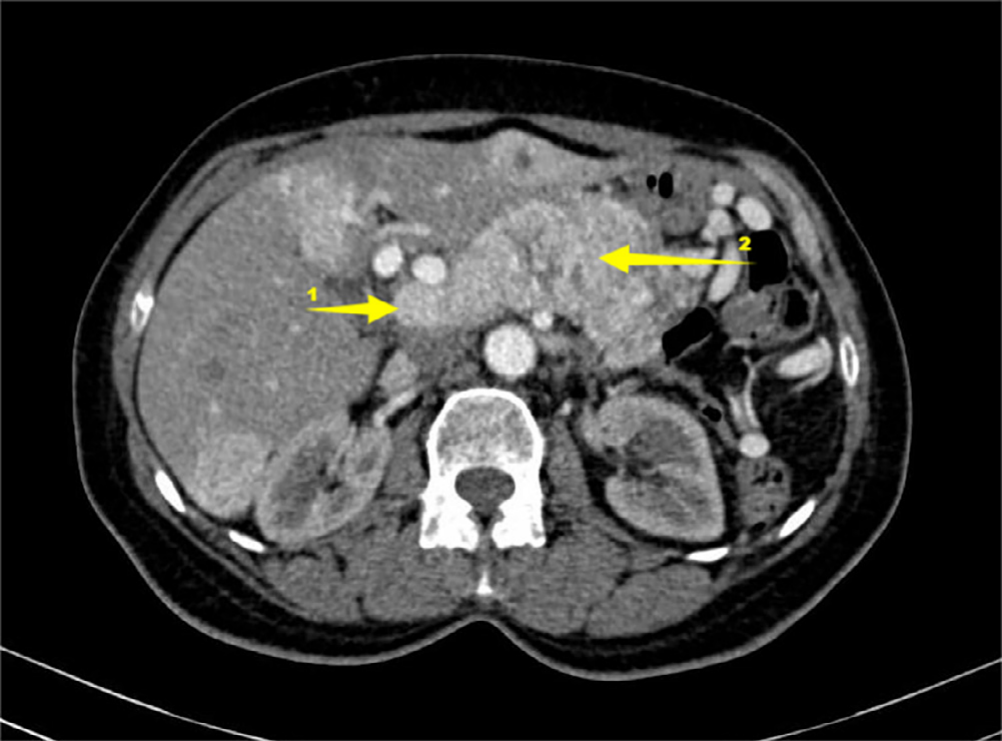
Abdominal injected CT on axial plane. 1 liver tumor. 2 tumoral thrombosis inside portal vein.

**Fig. 3:**
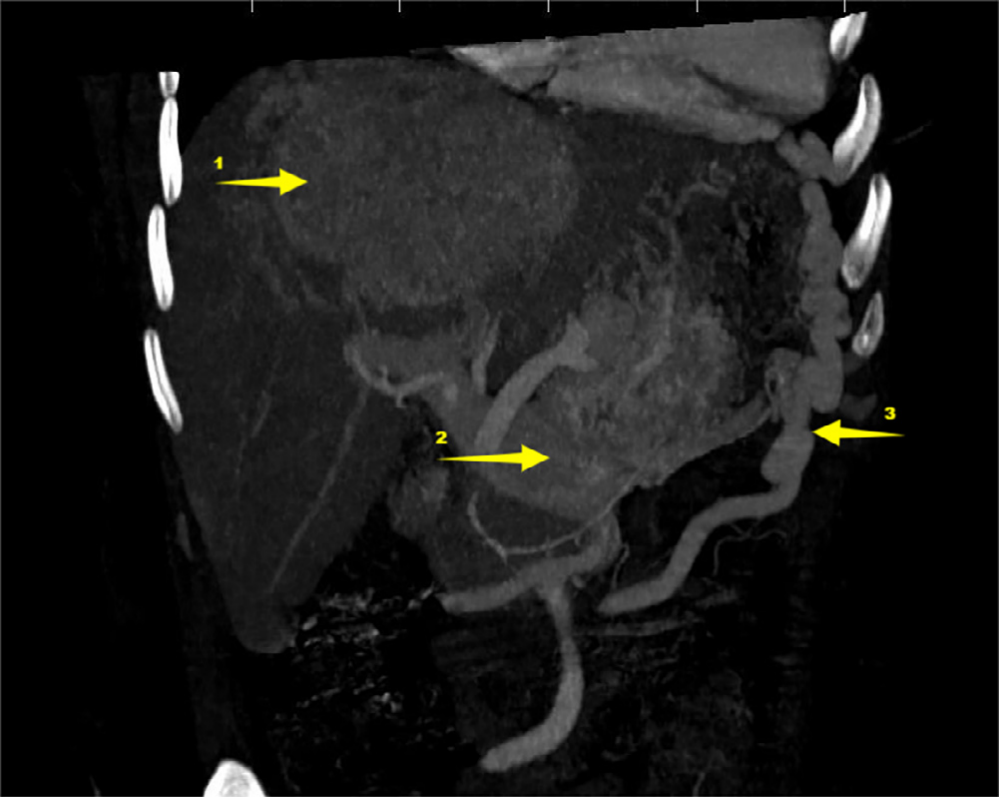
Abdominal injected CT on coronal plane with maximum intensity projection mode 1 liver tumor. 2 tumoral thrombosis inside portal vein 3 collateral circulation.

Additionally, a voluminous mass was identified within the splenic vein (Fig. 1, Fig. 2), extending into the portal trunk and causing extensive collateral venous circulation in the gastro-splenic region (Fig. 3). The mass demonstrated vivid arterial-phase enhancement, angiogenesis within the thrombus (“vessel-inside-vessel” appearance), and late-phase washout, mirroring the enhancement pattern of the hepatic lesions.

**Fig. 4:**
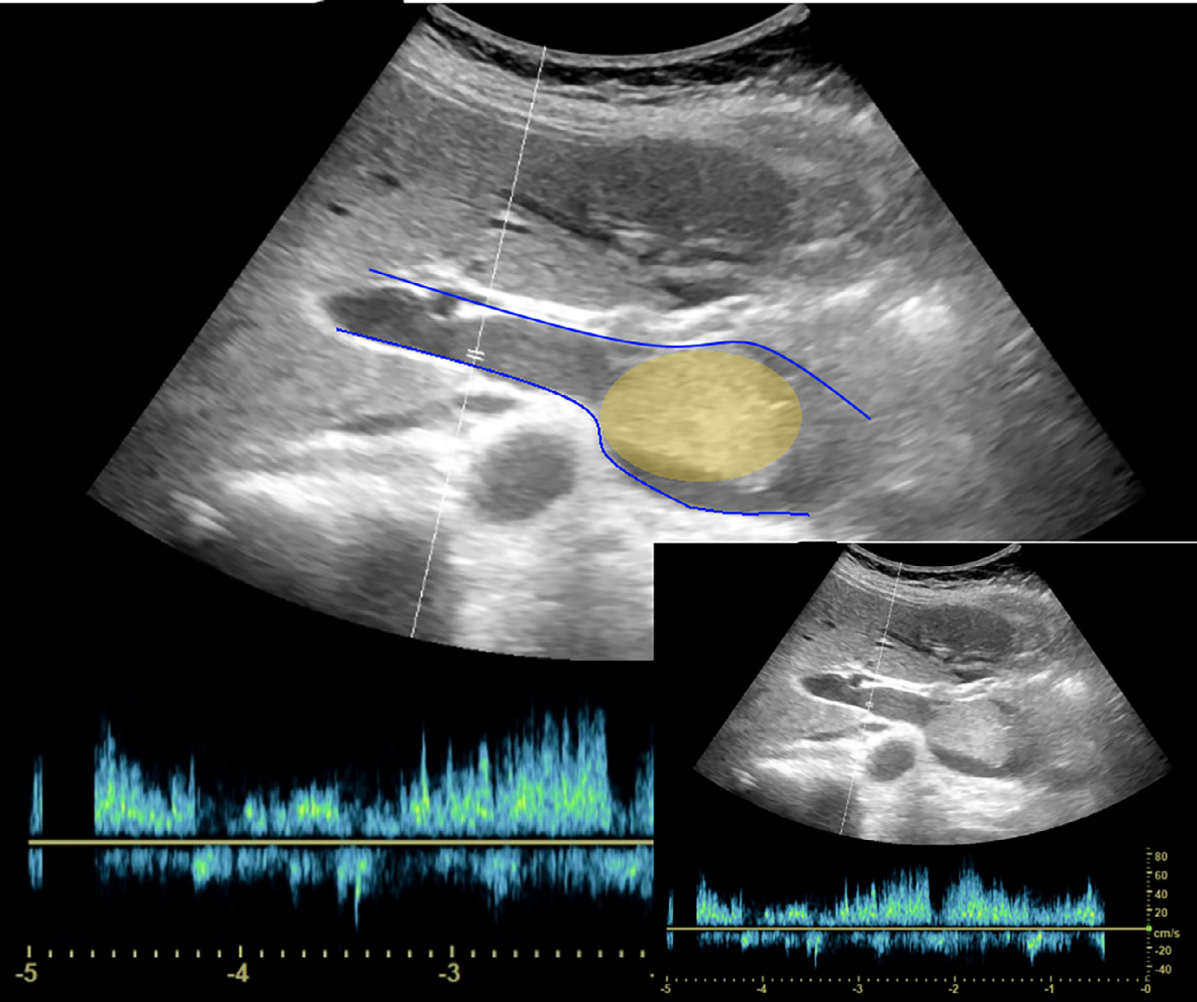
Ultrasound picture showing the thrombosis (yellow) inside the portal vein (blue).

**Fig. 5:**
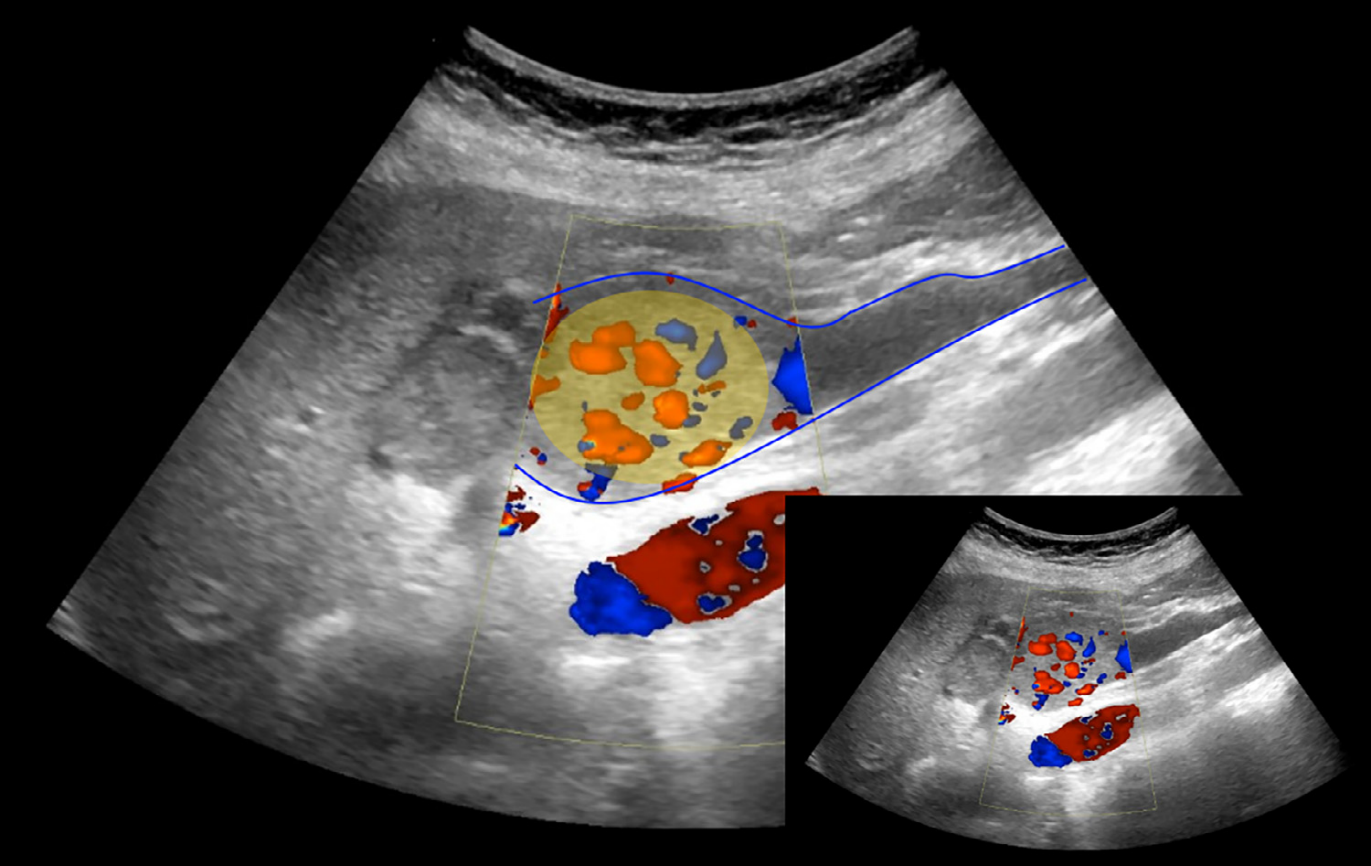
Ultrasound picture showing existence of vascularity inside the tumoral thrombosis (yellow).

Ultrasonography revealed a hypoechoic mass within the main portal trunk and splenic vein (Fig. 4). Color Doppler imaging showed hypervascularity (Fig. 5), with arterial and venous flow confirmed on spectral Doppler. A percutaneous ultrasound-guided biopsy was performed, and histopathological examination confirmed poorly differentiated hepatocellular carcinoma.

A multidisciplinary team meeting was held to evaluate therapeutic options. Given the advanced stage of hepatocellular carcinoma with endoportal tumoral thrombosis, curative treatment was not feasible. Consequently, the patient was started on palliative chemotherapy.

## Discussion

Carcinomas typically metastasize to distant organs through hematogenous spread, with cancer cells escaping the primary tumor and entering systemic circulation. Lung capillaries often serve as the first site of metastasis, followed by systemic arterial dissemination to distant organs. However, intravascular metastases from carcinomas are exceptionally rare.

Hepatocellular carcinoma is one of the most common malignancies globally. Interestingly, distant organ metastases are uncommon, even in advanced stages of HCC, with a predilection instead for intravascular and intrahepatic parenchymal spread [1].

Portal vein thrombosis (PVT) is a frequent and adverse manifestation of HCC, signifying a poor prognosis. It is observed in 10%-40% of HCC patients at diagnosis and 35%-44% at death or liver transplant. PVT is associated with metastatic disease, fewer treatment options, and reduced survival. HCC patients with PVT under supportive care have an overall survival of 2-4 months, compared to 10-24 months in those without PVT. Notably, thrombosis in the main portal vein predicts a poorer prognosis than involvement of a portal vein branch [2].

**Fig. 6:**
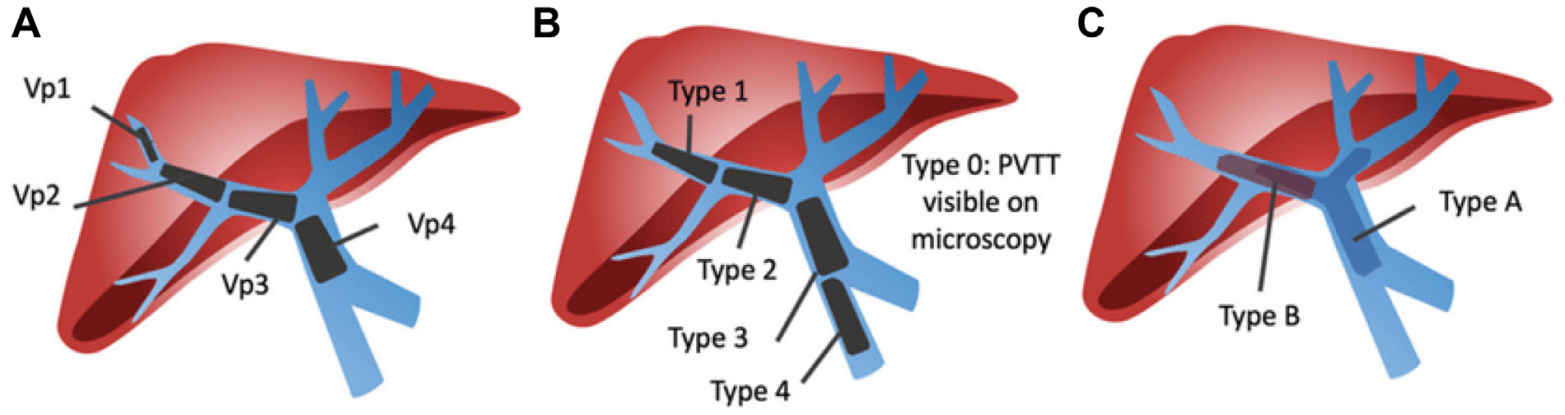
Portal vein tumor thrombosis classification systems. (A) Liver Cancer Study Group of Japan (B) Cheng classification (C) Xu classification.

Multiple classification systems have been proposed for HCC-associated portal vein tumor thrombus (PVTT):

• **X**u's Classification [3]:
  ○ Group A: Tumor thrombus involves the main portal vein trunk or both the right and left portal veins.
  ○ Group B: Tumor thrombus involves either the right or left portal vein branch.
• Liver Cancer Study Group of Japan (LCSGJ) Classification [4]:
  ○ VP0: No tumor thrombus in the portal vein.
  ○ VP1: Tumor thrombus distal to second-order branches of the portal vein.
  ○ VP2: Invasion of second-order branches of the portal vein.
  ○ VP3: Tumor thrombus in the first-order branch of the portal vein.
  ○ VP4: Tumor thrombus in the main trunk or contralateral branch of the portal vein.
• Shi's (Cheng's) Classification [5]:
  ○ Type IѲ: Microscopic portal invasion.
  ○ Type I: Tumor thrombus in segmental branches of the portal vein.
  ○ Type II: Tumor thrombus in either the right or left portal vein.
  ○ Type III: Tumor thrombus in the main portal vein.
  ○ Type IV: Tumor thrombus in the superior mesenteric vein (Fig. 6).

This case highlights a rare and extensive presentation of PVTT in HCC, characterized by angiogenesis within the thrombus, as demonstrated by the ``vessel-inside-vessel'' appearance on imaging. Understanding the classification and prognostic implications of PVTT is critical for tailored management strategies and improved patient outcomes.

## Patient consent

Written informed consent was obtained from the patient for the publication of this case report and accompanying images. The patient has reviewed the content and has provided permission for the use of their clinical data and imaging findings for educational and research purposes. All identifying information has been anonymized to protect the patient's privacy.
